# Midostaurin does not prolong cardiac repolarization defined in a thorough electrocardiogram trial in healthy volunteers

**DOI:** 10.1007/s00280-012-1825-y

**Published:** 2012-02-01

**Authors:** Adam del Corral, Catherine Dutreix, Alice Huntsman-Labed, Sebastien Lorenzo, Joel Morganroth, Robert Harrell, Yanfeng Wang

**Affiliations:** 1 Novartis Oncology, East Hanover, NJ USA; 2 Novartis Oncology, Basel, Switzerland; 3 ERT, East Bridgewater, NJ USA; 4 Osborne Research Center, LLC, Little Rock, AR USA; 5 Novartis Pharmaceuticals Corporation, 180 Park Avenue, Florham Park, NJ 07932 USA

**Keywords:** Clinical trials, Hematology, Pharmacokinetics and drug metabolism, Pharmaceutical medicine, Cardiovascular

## Abstract

**Purpose:**

Midostaurin (PKC412) is a multitargeted tyrosine kinase inhibitor of FMS-like tyrosine kinase 3 receptor (FLT3), c-KIT, and other receptors. Midostaurin is active in patients with acute myeloid leukemia and systemic mastocytosis. Although no substantive risk for cardiac abnormalities has been observed with midostaurin in clinical studies thus far, some TKIs have been shown to affect cardiac repolarization. Here we evaluated midostaurin’s effect on cardiac repolarization.

**Methods:**

This phase I study evaluated the effect of midostaurin (75 mg twice daily for 2 days; 75 mg once on day 3) on the heart rate–corrected QT (QTc) interval in a parallel design with active (moxifloxacin) and placebo control arms in healthy volunteers.

**Results:**

The maximum mean QTc change from baseline corrected using Fridericia’s correction (QTcF) for midostaurin compared with placebo was 0.7 ms at 24 h post dose on day 3. The highest upper bound of the 1-sided 95% CI was 4.7 ms, which excluded 10 ms, demonstrating a lack of QTcF prolongation effect. Assay sensitivity was demonstrated by modeling the moxifloxacin plasma concentration versus QTcF change from baseline, which showed a clear positive increase in QTcF with increasing moxifloxacin plasma concentrations, as expected based on previous studies. In the 4-day evaluation period, a minority of participants (34.6%) experienced an adverse event; 97.0% were grade 1. No grade 3 or 4 adverse events were reported.

**Conclusion:**

Midostaurin demonstrated a good safety profile in healthy volunteers, with no prolonged cardiac repolarization or other changes on the electrocardiogram.

**Electronic supplementary material:**

The online version of this article (doi:10.1007/s00280-012-1825-y) contains supplementary material, which is available to authorized users.

## Introduction

Midostaurin (PKC412; *N*-benzoylstaurosporin) is a multitargeted tyrosine kinase inhibitor (TKI) of several class III receptor tyrosine kinases with known roles in hematopoiesis and leukemia. These receptors include wild-type and mutant variants of the FMS-like tyrosine kinase 3 receptor (FLT3), c-KIT, platelet-derived growth factor receptor-β, and others [1]. Mutations leading to constitutive activation of FLT3, which is involved in regulating the proliferation, differentiation, and apoptosis of myeloid progenitors, occur in the blasts of about 30% of patients with acute myeloid leukemia (AML) [2–4], highlighting the potential utility of therapies targeting FLT3 in AML treatment. Furthermore, in vitro analysis of FLT3 inhibitors with varying levels of selectivity suggests that less-selective FLT3 inhibitors or those with broader tyrosine kinase inhibition profiles may offer a cytotoxic advantage in patients with newly diagnosed AML [5].

Midostaurin has demonstrated activity as a single agent [6], has induced complete remissions in combination with chemotherapy in patients with AML [7], and is currently under evaluation in a phase III registration trial in patients with newly diagnosed FLT3-mutant AML at a dose of 50 mg twice daily in combination with standard chemotherapy [8].

The inhibitory activity of midostaurin against c-KIT is also of interest because of the role that mutations in c-KIT play in aggressive systemic mastocytosis (ASM). Mutations in c-KIT are found in approximately 80% of patients with ASM [9]. Preliminary results of a multicenter, phase II study of midostaurin (100 mg twice daily) in 26 patients with ASM, mast cell leukemia, or systemic mastocytosis without an associated hematologic clonal nonmast cell lineage disease (AHNMD) demonstrated that patients achieved a high overall response rate of 69%, regardless of c-KIT mutation status [10]. A global phase II study was initiated to evaluate the efficacy and safety of midostaurin (100 mg twice daily until progressive disease, intolerability, or withdrawal) in patients with ASM or mast cell leukemia with or without an AHNMD [11].

The plasma concentrations of midostaurin and its metabolite CGP62221 accumulate in a time-linear manner in the first 3–5 days of daily oral dosing [12]. Thereafter, the pharmacokinetics (PK) become nonlinear, with a large increase in bioavailability between day 5 and day 28 to reach a new “pseudo steady state.” In contrast, the longer-lasting hydroxymetabolite CGP52421 continues to accumulate to reach approximately seven times the concentration of midostaurin and CGP62221 at steady state. Biliary excretion is the major pathway for elimination of midostaurin, CGP62221, and CGP52421.

Some TKIs have been shown to affect cardiac repolarization, as detected by heart rate–corrected QT (QTc) prolongation [13–18]. Although no previous clinical studies have suggested a substantive risk for cardiac abnormalities with midostaurin, a dedicated study has not been conducted to investigate the possible effects of midostaurin on the QTc corrected using Fridericia’s correction (QTcF) interval. Described here are the results from a randomized study using placebo and active control arms to determine whether midostaurin administered at a dose of 75 mg twice daily for 2 days and 75 mg once daily for 1 day affects QTcF intervals in healthy adult volunteers.

## Methods

### Patients

Inclusion criteria for healthy volunteers, aged 18–45 years, included no clinically significant deviations from normal in medical history, physical examination, vital signs, or clinical laboratory determinations. A body weight between 50 and 100 kg and a body mass index between 18 and 33 kg/m^2^ were also required. Exclusion criteria included, but were not limited to, a history or family history of long QT-interval syndrome, heart disease, and any other severe or uncontrolled medical or psychiatric condition. Smoking and drug and/or alcohol abuse within 30 days of randomization, use of prescription drugs within 14 days of randomization, and use of CYP3A4 enzyme-inducing or enzyme-inhibiting agents within 4 weeks of dosing were prohibited. Participants were discontinued if they had abnormal electrocardiogram (ECG) results on day −1, during placebo run-in, and were followed until resolution of abnormality. These participants were replaced, as is standard in QTc studies, to ensure that a sufficient number of participants were evaluable for the ECG analysis.

Participants randomized into 1 of the 3 arms of the study were evaluated as the randomized set, regardless of whether they ever received study medication. The safety population consisted of all participants who received at least 1 dose of study medication. The PK set consisted of all participants who completed at least 1 dose of midostaurin or moxifloxacin and had evaluable PK profiles on day 1 and/or day 3. The ECG set consisted of participants who completed all scheduled doses of study medication from day 1 to day 3 and had an available baseline ECG measurement and at least 1 ECG measurement on day 3.

### Study design

This study was a phase I, randomized, double-blind, placebo- and active-controlled, 3-way, parallel-group study conducted at a single center in the United States and approved by the Arkansas Research Medical Testing, LLC, Institutional Review Board. The design followed the recommendations of the International Conference on Harmonisation of Technical Requirements for Registration of Pharmaceuticals for Human Use (ICH) E14 guideline on “The Clinical Evaluation of QT/QTc Interval Prolongation and Proarrhythmic Potential for Non-Antiarrhythmic Drugs” [19]. As such, the trial included a concurrent positive control group and addressed intrinsic variability by conducting multiple ECGs at baseline and during the study. The study was conducted according to the ethical principles of the Declaration of Helsinki, and written informed consent was obtained from each subject during screening.

**Fig. 1:**
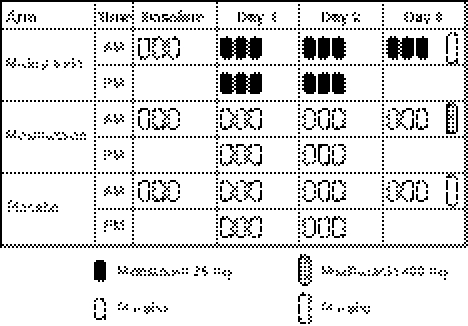
Study drug administration per treatment arm

Participants were randomized to 1 of 3 treatment arms: midostaurin administered orally at 75 mg twice daily on days 1 and 2 and a single dose on day 3, moxifloxacin administered orally at a single 400-mg dose on day 3, or placebo (Fig. 1). The treatment regimen was selected to achieve maximal plasma exposure for QTc evaluation while minimizing the risks of excessive or prolonged exposure in healthy volunteers. In a prior study, patients with diabetes mellitus treated with multiple oral doses of midostaurin for 28 days at 4 dose levels (25 mg twice daily, 50 mg twice daily, 75 mg twice daily, and 75 mg 3 times daily) as well as a single oral 100-mg dose, demonstrated a marked increase in frequency of adverse events at doses above the 75-mg twice-daily dose [20]. Thus, the 75-mg twice-daily dose was expected to be safe and effective and was associated with a midostaurin cumulative *C*_max_ similar to that observed with a 50-mg twice-daily dose of midostaurin in a phase Ib study of patients with newly diagnosed AML treated with various doses of midostaurin [7].

Moxifloxacin (400 mg) was used in the active control arm because this agent is known to prolong QTc intervals in a dose-dependent manner [21]. The difference in QTcF interval prolongation between the moxifloxacin and placebo arms served as an indicator of assay sensitivity. Moxifloxacin was overencapsulated to make the capsules visually identical to the placebo. Novartis supplied capsules for blinding of the active control via overencapsulation by the pharmacy of the site. Dissolution testing was performed on moxifloxacin overencapsulated tablets, and they were determined to have equivalent dissolution to regular moxifloxacin.

Because the metabolite CGP52421 exhibits a very long half-life (up to 1 month) in human participants, a parallel study design was used instead of a crossover design to avoid the potential carryover effect of this analyte. This study did not evaluate the effect of CGP52421, which would require 21–28 days of treatment to reach the steady state, because of ethical and safety concerns related to long-term exposure to a drug in healthy volunteers. Study drugs were administered at 8:00 AM and 8:00 PM; breakfast and dinner were provided at 10:00 AM and 5:30 PM, respectively.

The primary objective of this study was to determine the effect of multiple doses of midostaurin on the QTcF interval. The primary variable evaluated was the change from baseline (day −1) in the QTcF interval over the protocol-defined time points on day 3 with midostaurin. The baseline comparison was from day −1 to day 3 at matched time points. The secondary objectives were safety, tolerability, cardiac intervals (QT, QTcB [corrected using Bazett’s correction], QTcI [individually corrected], length of QRS complex of waves, interval between RR waves [RR], interval between PR waves [PR]), and heart rate following multiple doses of midostaurin.

### Statistical methods

To claim a lack of effect of multiple doses of midostaurin on QTcF interval, the following hypothesis was tested predose (0 h) and at all 8 post dose time points on day 3:

$$ {\text{H}}_{0} :{\text{U}}\{ \mu_{mido(t)} - \mu_{placebo(t)} \} \ge 10,t = 0,0. 5,{ 1},{ 2},{ 3},{ 4},{ 8},{ 12},{\text{ and 24 hours}}\;{\text{versus}} $$

$$ {\text{H}}_{ 1} : \cap \{ \mu_{mido(t)} - \mu_{placebo(t)} \} \, < 10,t = \, 0, \, 0. 5,{ 1},{ 2},{ 3},{ 4},{ 8},{ 12},{\text{ and 24 hours}} $$

where μ_*mido*(*t*)_ and μ_*placebo*(*t*)_ are the mean QTcF changes from baseline observed following all scheduled doses of midostaurin and placebo, respectively, at time point *t* on day 3. The lack of QT effect for midostaurin was established if the null hypothesis was rejected. The null hypothesis was rejected if the highest upper bound of the 95% 1-sided confidence interval (CI) for the time-matched mean effect (adjusted for baseline and placebo) of midostaurin on the QTcF interval at all time points excluded 10 ms.

The following hypothesis was tested to confirm that the study had sufficient assay sensitivity:

$$ {\text{H}}_{0} : \cap \{ \mu_{moxi(t)} - \mu_{placebo(t)} \} \, \le 5,t = \, 0. 5,{ 1},{ 2},{ 3},{\text{ and 4 hours}}\;{\text{versus}} $$

$$ {\text{H}}_{ 1} :{\text{U}}\{ \mu_{moxi(t)} - \mu_{placebo(t)} \} \, > 5,t = \, 0. 5,{ 1},{ 2},{ 3},{\text{ and}}\, 4\,{\text{hours}} $$

where μ_*moxi*(*t*)_ was the mean QTcF change from baseline observed following moxifloxacin 400 mg at time point *t*. If the null hypothesis was rejected, the study had sufficient sensitivity. The Simes method was used for the protection of an experiment-wise 5% type I error [22, 23]. With the Simes method, the original *P* values corresponding to 0.5, 1, 2, 3, and 4 h post baseline are ordered increasingly, that is, *P*_1_ ≤ *P*_2_ ≤ *P*_3_ ≤ *P*_4_ ≤ *P*_5_. After the Simes correction, the *P* values were 5*P*_1_, 5*P*_2_/2, 5*P*_3_/3, 5*P*_4_/4, and *P*_5_ respectively. If any of the 5 adjusted *P* values were <.05, assay sensitivity was claimed. Only the participants who completed all scheduled doses of study medication from day 1 to day 3 and had at least 1 ECG on day −1 (baseline) and at least 1 ECG on day 3 (time-matched day 3 data for the time-matched analysis) were included in the assay sensitivity test.

Electrocardiogram measurements at each time point were calculated as an average of 3 separate ECG extractions or replicates. (Each extraction was the mean of 3 beats.) If fewer than 3 measurements were available, the available samples were averaged (i.e., a minimum of 1 measure was required). For each subject, the time-matched baseline value was subtracted from the QT/QTc intervals to determine the change from baseline in QT/QTc intervals for that subject. The 2 null hypotheses described above were tested in a linear mixed-effect model with a compound symmetry covariance structure. The model included the baseline measure as covariate and treatment, time, and the treatment-by-time interaction as fixed effects, where time was a categorical variable and subject was a random effect.

The time-matched analysis was conducted on the QTcF change from the time-matched baseline as recommended by the ICH E14 guideline [19]. Although modeling change from the time-matched baseline was the primary analysis, the change from the time-averaged baseline was also analyzed using the same model. For the averaged baseline, each triplicate ECG collection was averaged first, and then the averaged baseline was calculated based on all the averaged triplicate ECG and unscheduled ECGs.

Exploratory analyses were performed to characterize the relationship between drug concentrations and changes in QT intervals to assist with interpretation of the study results. A linear random-effects model was fit to the QTcF/QTcB/QTcI/QT change from day −1 (baseline) to day 3 and concentration data for midostaurin or its 2 metabolites (CGP52421 and CGP62221) or moxifloxacin. Baseline QTcF was included in the model as a covariate. The QTcF effect and its upper 1-sided 95% CI were computed at the 25% quartile, mean, 75% quartile, and median of the *C*_max_ for midostaurin or its 2 metabolites or moxifloxacin. This exploratory analysis was applied to both the change from the time-matched baseline and the change from time-averaged baseline.

Outlier analysis for QTc was also exploratory because this study was not powered to detect individuals with genetic sensitivity to potential QT-prolonging drugs. The nonspecific outlier criterion was a change from baseline in QTc interval of 30–60 ms.

### Clinical assessments

Standard triplicate 12-lead ECGs were obtained at 9 time points over 24 h at baseline on day 3 and at 2 time points on day 1. Electrocardiogram analysis was performed at a blinded central reading facility (ERT, East Bridgewater, NJ) in digital format, with paper tracings obtained and archived immediately on site. Vital signs were assessed daily. Clinical laboratory parameters were assessed at baseline and at the end of study. Self-reported adverse events were continuously recorded from the first study treatment (placebo, day −1) through the end of study on day 4.

### Pharmacokinetic and pharmacodynamic assessments

Blood samples for PK analysis were collected predose and 0.5, 1, 2, 3, 4, 8, 12, and 24 h post dose on days 1 and 3 at the same time as ECG assessments. Moxifloxacin, midostaurin, CGP62221, and CGP52421 concentrations were determined by high-performance liquid chromatography/mass spectrometry with a limit of quantification of 50 and 10 ng/mL respectively. Noncompartmental analysis (WinNonlin™ version 5.2, Pharsight, Sunnyvale, California) was performed to determine the following PK parameters: *C*_max_, *T*_max_, minimum (trough) plasma concentration over a dosing interval (*C*_min_), and AUC calculated using a trapezoidal method. For moxifloxacin, the AUC from time 0 to the last measurable concentration sampling time was calculated (AUC_0–tlast_). For midostaurin and its metabolites, the AUC from time 0 to 12 h (AUC_0–12h_) was calculated following the first dose on day 1, and the AUC from 0 to 24 h (AUC_0–24h_) was calculated on day 3. The relationship between drug concentration and change in QT interval was explored to assist with interpretation of the results.

## Results

Demographic parameters were well-distributed among the study arms (Supplementary Table 1). A total of 192 healthy volunteers completed the study, and 161 were considered eligible for analysis of the primary endpoint (ECG set; *n* = 54 in the midostaurin arm, 64 in the placebo arm, and 43 in the moxifloxacin arm). In the midostaurin arm, 24 participants discontinued the study (Table 1): 19 because of adverse events, predominantly gastrointestinal events of vomiting (*n* = 17) and 2 events of grade 1 tachycardia during the placebo run-in period (i.e., before active treatment). All instances of vomiting occurred within 4 h of dosing, and patients who experienced vomiting within 4 h of dosing were ineligible for the ECG set. Because data from patients who vomited could not be used for the primary objective, these patients were discontinued immediately from the trial. No participants in the other treatment groups discontinued because of adverse events. Sixteen replacement participants were also enrolled to ensure that a sufficient number of participants were evaluable for the ECG analysis.

**Table 1:** Subject disposition (Randomized set)

	Midostaurin (*n* = 80)	Moxifloxacin (*n* = 44)	Placebo (*n* = 68)
*Participants, no. (%)*			
Completed	56 (70.0)	44 (100.0)	66 (97.1)
ECG set	54 (67.5)	43 (97.7)	64 (94.1)
Discontinued	24 (30.0)	0 (0.0)	2 (2.9)
*Main cause of discontinuation*			
Death	0 (0.0)	0 (0.0)	0 (0.0)
Adverse event(s)	19 (23.8)	0 (0.0)	0 (0.0)
Abnormal test procedure	2 (2.5)	0 (0.0)	0 (0.0)
Withdrew consent	2 (2.5)	0 (0.0)	1 (1.5)
Protocol violation(s)	1 (1.3)	0 (0.0)	0 (0.0)
Administrative reasons	0 (0.0)	0 (0.0)	1 (1.5)

ECG, electrocardiogram

### ECG analysis

**Fig. 2:**
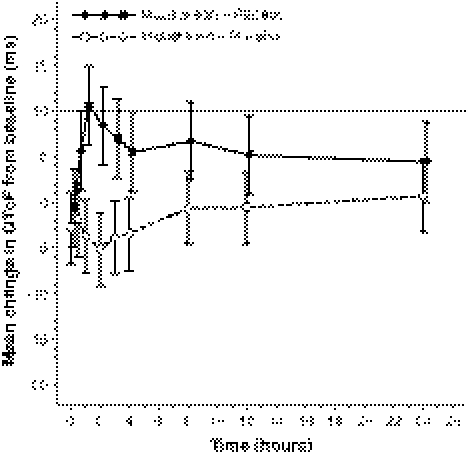
Estimated mean change (95% 1-sided CI boundaries indicated by bars) from time-matched baseline in heart rate–corrected QT intervals using Fridericia’s correction (QTcF) on day 3 compared with placebo (electrocardiogram data set). *QTcF*, heart rate–corrected QT interval corrected using Fridericia’s correction

For the midostaurin treatment arm, the upper bounds of the 1-sided 95% CI for the estimated QTcF change from time-matched baseline (compared with placebo; delta–delta analysis) for all 9 time points on day 3 compared with placebo were <10 ms (Fig. 2). The maximum mean change from baseline for midostaurin compared with placebo occurred 24 h post dose on day 3 and was 0.7 ms; its highest upper bound of the 1-sided 95% CI was 4.7 ms, which excluded 10 ms (Supplementary Table 2). Thus, midostaurin did not demonstrate the potential for proarrhythmic effects associated with QT interval prolongation.

**Fig. 3:**
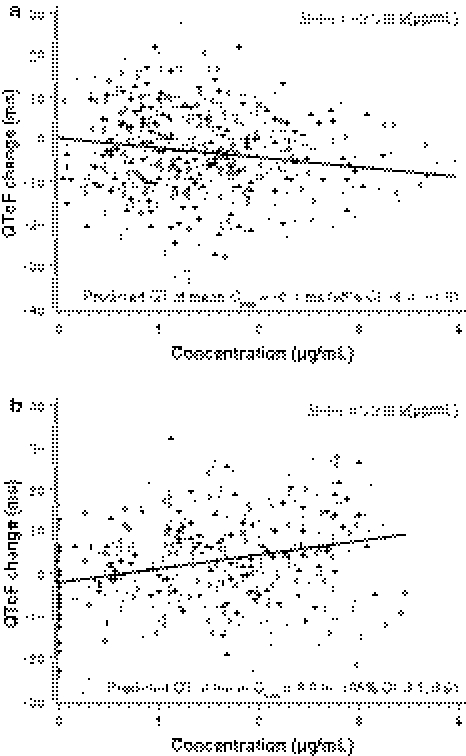
Plasma concentrations of midostaurin (**a**) and moxifloxacin (**b**) versus change from time-matched baseline in heart rate–corrected QT intervals using Fridericia’s correction (QTcF) on day 3 (electrocardiogram data set). The line represents the fixed effect of the fitted concentration–QTcF model. CI, confidence interval; C_max_, maximum plasma concentration; QTcF, heart rate–corrected QT interval corrected using Fridericia’s correction

Consistent with time-matched analysis, the QTcF change from time-averaged baseline demonstrated a lack of effect on QTc prolongation. The maximum mean change from baseline for midostaurin compared with placebo was 2.5 ms and occurred 24 h post dose on day 3. The highest upper bound of its 95% CI was 4.9 ms. A negative or nonsignificant concentration versus QTcF slope was observed for midostaurin (Fig. 3a), CGP62221, and CGP52421 concentrations (−2.3, −3.3, and 0.2, respectively), confirming no QT prolongation at the administered dose.

The active control moxifloxacin had a maximum mean QTcF prolongation from time-matched baseline compared with placebo of 10.7 ms, which occurred 1 h post dose on day 3 (Fig. 2). The lower bound of the 1-sided 95% CI of 6.4 ms exceeded 5 ms (original *P* = .015), demonstrating QT prolongation for moxifloxacin. However, when the correction of Simes was applied to adjust for multiple comparisons, there were no statistically significant changes in QTcF interval from baseline at the 5 time points (*P* = 0.07 at 1 h post dose). At 0.5, 2, 3, and 8 h, moxifloxacin had a maximum mean QTcF prolongation from time-matched baseline of between 5 and 10 ms, with the upper limit of the 95% CI between 10 and 15 ms (14.9 ms at 1 h post dose).

Using time-averaged baseline, the maximum mean change from baseline for the moxifloxacin arm compared with placebo occurred 1 h post dose on day 3 and was 10.2 ms. The lower bound of its 95% CI was 7.6 ms (*P* = 0.003 at 1 h post dose, adjusted for multiple comparisons). Unlike with midostaurin and its metabolites, there was a clear positive slope of QT change from baseline with increasing plasma moxifloxacin concentrations (4.0 for QT, 2.6 for QTcB, 3.2 for QTcF) that was statistically significant (Fig. 3b).

QTcB changes in the 30- to 60-ms category were detected in 1 (1.3%) subject in the midostaurin arm, 7 (15.9%) participants in the moxifloxacin arm, and 1 (1.5%) subject in the placebo arm in the exploratory outlier analyses (Table 2). QTcB results between 450 and 480 ms post baseline were also detected in 1 (1.3%) subject in the midostaurin arm and in 1 (2.3%) subject in the moxifloxacin arm. No subject had a QTc duration >480 ms or a change from baseline in QTc >60 ms. QRS, RR, and PR abnormalities were uncommon in all arms (Table 2).

**Table 2:** Participants with notable QTc intervals and other electrocardiogram parameters (Safety set)

	Midostaurin (*n* = 79)	Moxifloxacin (*n* = 44)	Placebo (*n* = 68)
*QTcF, no. (%)*			
>30 ms^a^	0	0	0
>60 ms^a^	0	0	0
New >450 ms^b^	0	2/44 (4.5)	0
*QTcB, no. (%)*			
>30 ms^a^	1 (1.3)	7 (15.9)	1 (1.5)
>60 ms^a^	0	0	0
New >450 ms^b^	1/78 (1.3)	1/44 (2.3)	0
*QTcI, no. (%)*			
>30 ms^a^	0	0	0
>60 ms^a^	0	0	0
New >450 ms^b^	1/79 (1.3)	2/44 (4.5)	0
QRS increase ≥25% and resultant QRS >100 ms	0	0	0
RR increase ≥25% and resultant RR >1,200 ms	1 (1.3)	0	1 (1.5)
RR decrease ≥25% and resultant RR <600 ms	1 (1.3)	0	0
PR increase ≥25% and resultant PR >200 ms	0	0	0

*QTcF*, heart rate–corrected QT interval (QTc) corrected using Fridericia’s correction; *QTcB, * QTc corrected using Bazett’s correction; *QTcI, * QTc individually corrected; *QRS*, length of QRS complex of waves; *RR*, interval between RR waves; *PR*, interval between PR waves

^a^Time-matched change from baseline

^b^Number of post baseline values >450 ms/number of baseline values >450 ms

No symptomatic, new post baseline morphologic waveform changes on the 12-lead ECG were identified in the study. Three participants in the midostaurin arm had new post baseline T-wave abnormalities at a single time point or as a single occurrence, but these abnormalities were deemed by the investigator not to be clinically significant. However, 1 and 4 participants in the placebo and moxifloxacin arms, respectively, experienced new post baseline T-wave abnormalities, some at multiple time points. No new U-wave abnormalities were noted in the placebo or midostaurin arm, but 1 case was identified in the moxifloxacin arm.

### Pharmacokinetics

**Fig. 4:**
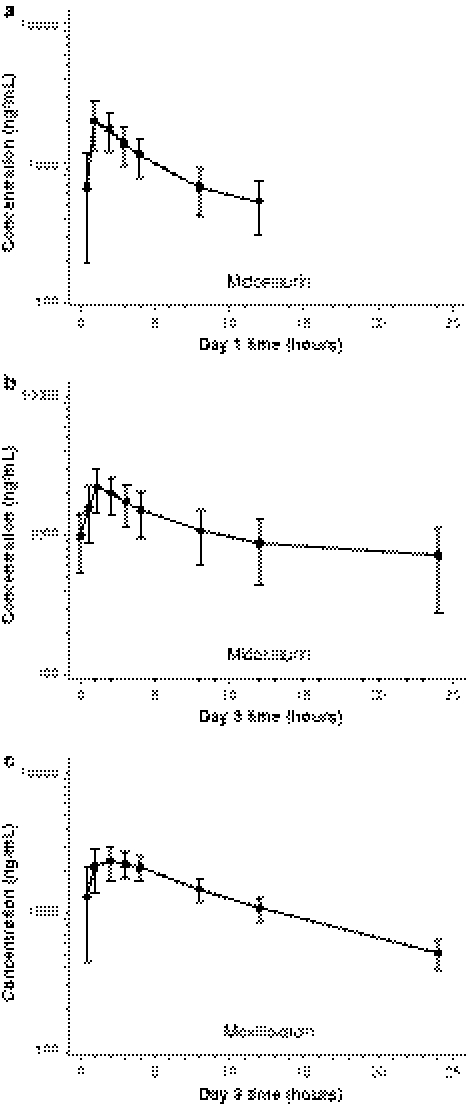
Mean (standard deviation indicated by bars) concentration–time profiles for midostaurin on day 1 (**a**) and day 3 (**b**) and for moxifloxacin on day 3 (**c**)

Peak concentrations of midostaurin (Supplementary Table 3) were observed mainly at 1 h post dose on both days 1 and 3 (Fig. 4) and of CGP62221 and CGP52421 at 3–4 h post dose. The mean *C*_max_ for moxifloxacin was 2544.2 ng/mL (standard deviation [SD] = 495.0) and occurred at a median of 2.1 h (range, 0.6–4.1 h) after administration (Supplementary Table 4). The mean AUC_0–tlast_ of moxifloxacin was 29 407.9 ng*h/mL (SD = 5165.6 ng*h/mL).

### Safety

In total, 66 (34.6%) participants experienced adverse events on the study drug (Supplementary Table 5). These adverse events were generally mild and transient, with no grade 3 or 4 events reported. Of the total adverse events reported, 97.0% were grade 1. Four grade 2 events were reported: headache (1 participant), nausea (2 participants, 1 of whom discontinued because of vomiting), and diarrhea (1 participant). Gastrointestinal adverse events were more common in the midostaurin arm, as expected for this population and drug class. Two participants in the midostaurin arm experienced grade 1 tachycardia during the placebo run-in period (day −1) and were discontinued prior to treatment with midostaurin. They were both followed until resolution of symptoms. No other cardiac events were reported in any participants. All incidences of vomiting occurred within 4 h of midostaurin dosing, and these patients were not included in the ECG or PK data analysis. There were no clinically relevant changes or adverse events related to laboratory values or vital signs in any treatment group.

## Discussion

Because some TKIs exert unexpected pharmacologic effects on cardiac repolarization, the current thorough QT/QTc study was designed to assess the cardiac interval effects of midostaurin in healthy participants. In particular, FLT3 is recognized as a major target in the treatment of AML, and agents specifically designed to target this receptor, including AC220 [16] and MLN518 [17], have been shown to induce prolongation of the QT interval in clinical trials, as has the multikinase inhibitor sorafenib [18].

In this study, we demonstrated that midostaurin, an inhibitor of FLT3, c-KIT, and other tyrosine kinases with established efficacy in patients with AML [6, 7] and ASM [10, 24], was not associated with prolonged cardiac repolarization or its related proarrhythmic effects. In a time-matched analysis for QTcF, midostaurin had no or minimal effect on the QT interval, with an upper bound of the 95% CI for QTcF values corrected for both baseline and placebo <5 ms. The threshold level of regulatory concern, as established in the ICH E14 guideline [19], is a 10-ms mean increase in QTc as the upper bound of the 95% CI. The results for the time-averaged analysis were consistent with those determined using the time-matched analysis.

Despite historical reliance on the QTc change from baseline for determining a drug’s proarrhythmic risk, the importance of the concentration–QT relationship in interpreting thorough QT studies is increasingly being realized [25, 26]. Concentration–QTcF slopes for midostaurin and its metabolites CGP62221 and CPG52421 were either negative or not statistically significant, which further supports the lack of prolonged cardiac repolarization with midostaurin. Furthermore, the placebo arm’s mean QTcF change from baseline was within 5 ms, demonstrating that spontaneous factors were very well controlled.

On the basis of previous studies, the expected effect of the active control moxifloxacin on the QTcF interval was 8–13 ms [27]. Our results were consistent with this finding, with the lower CI >5 ms at hour 1 post administration, QTcF increases between 5 and 10 ms at 0.5, 2, 3, and 8 h post administration, and upper CIs between 10 and 15 ms with moxifloxacin treatment. In the time-matched analysis, these increases were not significantly different from placebo after multiple time point correction methods to establish the sensitivity of the assay. In a time-averaged analysis, however, the maximum mean change from baseline in the moxifloxacin arm compared with the placebo arm was significant. The lack of significance in the time-matched analysis may be related to the slightly lower moxifloxacin *C*_max_ observed in this study (2544 ng/mL) compared with what has been previously reported (2830 ng/mL) [28]. The PK profile of moxifloxacin was somewhat flattened, which was most likely due to overencapsulation [29].

Linear regression analyses showed a statistically significant positive slope of QT change from baseline with increasing moxifloxacin plasma concentrations. The moxifloxacin slope for QTcF (3.2 ms per μg/mL) was consistent with those found in 5 other thorough QTc studies, in which the mean slope estimates were 2.5, 2.4, 3.3, 3.5, and 4.3 ms per μg/mL [25, 26]. This positive slope, and the fact that moxifloxacin concentrations reached levels expected for overencapsulation [29], established the sensitivity of the assay. These findings support the value of determining the slope of the QT-concentration curve when overencapsulation is used for a double-blinded positive control.

Electrocardiogram analysis demonstrated that midostaurin had no effects on heart rate, atrioventricular conduction, or cardiac depolarization, as measured by the PR and QRS interval durations. No participants in any group met the specific outlier criteria for U-wave or QTc interval, although the analysis was exploratory. No QTcF, QTcB, or QTcI changes from baseline >60 ms or results >480 ms were detected in any arm. Overall, these data indicate that no clinically relevant changes in ECG parameters were detected in the midostaurin arm.

The adverse events experienced in this trial were mainly mild (97% were grade 1), and the gastrointestinal events in the midostaurin arm were expected and occurred at a lower frequency than has been observed previously in patients with AML treated with midostaurin [6]. Overall, midostaurin at a dose of 75 mg twice daily was safe and generally well tolerated in these healthy participants in a 4-day evaluation period.

The results of the concentration–QTcF regression analysis showed no evidence that midostaurin or its metabolite CGP62221 affected QTc duration, whereas the positive control moxifloxacin demonstrated the expected relationship between its concentration and the change in QTc. Despite the lack of prolonged cardiac repolarization with midostaurin in this carefully conducted study, we recommend continued ECG monitoring in clinical trials, but at a reduced frequency, as the QT effects of the long-lasting metabolite CGP52421 were not fully addressed in this relatively short study with a 4-day evaluation period.

## Electronic supplementary material

Below is the link to the electronic supplementary material.

Supplementary material 1 (DOC 148 kb)
