# Supplementary material for: Midostaurin does not prolong cardiac repolarization defined in a thorough electrocardiogram trial in healthy volunteers
Source: Cancer Chemother Pharmacol. 2012 Feb 1;69(5):1255–63. doi: 10.1007/s00280-012-1825-y (PMC3337405; doi:10.1007/s00280-012-1825-y)
Supplement: Supplementary file 1 — Supplementary material 1 (DOC 148 kb) [file 280_2012_1825_MOESM1_ESM.doc]

Midostaurin Does Not Prolong Cardiac Repolarization Defined in a Thorough Electrocardiogram Trial in Healthy Volunteers

*Cancer Chemotherapy and Pharmacology*

*Adam del Corral, PharmD^*^, Catherine Dutreix, PharmD, Alice Huntsman-Labed, PhD, Sebastien Lorenzo, MS, Joel Morganroth, MD, Robert Harrell, MD, and Yanfeng Wang, PhD*

*Corresponding author:

Adam del Corral, PharmD

Novartis Oncology, East Hanover, NJ

E-mail: Adam.delCorral@novartis.com.

**File contains Supplementary Tables**

**Supplementary Table 1** Subject Demographics and Baseline Characteristics (Randomized Set)

|  | Midostaurin (n = 80) | Moxifloxacin (n = 44) | Placebo (n = 68) | All Participants (N = 192) |
| --- | --- | --- | --- | --- |
| Age, years |  |  |  |  |
| Median | 28 | 31 | 30 | 29 |
| Range | 18-45 | 18-45 | 18-45 | 18-45 |
| Sex, no. (%) |  |  |  |  |
| Male | 53 (66.3) | 32 (72.7) | 51 (75.0) | 136 (70.8) |
| Female | 27 (33.8) | 12 (27.3) | 17 (25.0) | 56 (29.2) |
| Race, no. (%) |  |  |  |  |
| White | 20 (25.0) | 12 (27.3) | 19 (27.9) | 51 (26.6) |
| Black | 60 (75.0) | 32 (72.7) | 48 (70.6) | 140 (72.9) |
| Asian | 0 (0.0) | 0 (0.0) | 1 (1.5) | 1 (0.5) |
| Weight, kg |  |  |  |  |
| Median | 78.64 | 75.95 | 75.70 | 76.60 |
| Range | 52-100 | 55-100 | 54-100 | 52-100 |
| Height, cm |  |  |  |  |
| Median | 173.5 | 175.0 | 176.0 | 175.0 |
| Range | 152-193 | 155-193 | 152-192 | 152-193 |
| BMI, kg/m^2^ |  |  |  |  |
| Median | 26.0 | 25.2 | 25.1 | 25.6 |
| Range | 19-35 | 19-33 | 19-34 | 19-35 |

BMI, body mass index.

**Supplementary Table 2** QTcF Change on Day 3 From Time-Matched Baseline in Patients Treated With Midostaurin or Placebo (Electrocardiogram Set)

| **Time point**  **(h)^a^** | **Midostaurin** | **Placebo** | **Difference**  **(Midostaurin – Placebo)** | **Upper bound of the one-sided 95% CI** |
| --- | --- | --- | --- | --- |
| 0 | -3.2 | -0.4 | -2.7 | 1.3 |
| 0.5 | -5.2 | -3.3 | -1.9 | 2.1 |
| 1 | -6.3 | -2.6 | -3.7 | 0.3 |
| 2 | -6.2 | -1.2 | -5.1 | -1.1 |
| 3 | -5.2 | -1.4 | -3.9 | 0.1 |
| 4 | -6.4 | -3.0 | -3.4 | 0.6 |
| 8 | -1.9 | -1.2 | -0.7 | 3.3 |
| 12 | -3.1 | -2.5 | -0.6 | 3.4 |
| 24 | -3.9 | -4.6 | 0.7 | 4.7 |

^a^ Hours after administration.

###### Supplementary Table 3 Pharmacokinetic Parameters of Midostaurin and Its Metabolites After a Single Oral Dose of 75 mg on Days 1 and 3 (Pharmacokinetic Set)

| Day 1 (single dose) | T_max_ (hours) | C_max_ (ng/mL) | AUC_0-12h_ (ng * hour/mL) |
| --- | --- | --- | --- |
| Midostaurin (n = 70) | 1.0 (1.0-3.1) | 2142.1 (690.1) | 11 662.6 (3723.7) |
| CGP62221 (n = 70) | 3.1 (2.1-4.0) | 866.3 (169.2) | 7848.2 (1579.9) |
| CGP52421 (n = 67) | 4.0 (2.1-4.0) | 384.5 (65.1) | 3509.2 (609.8) |
| Day 3 (twice daily) | T_max_ (hours) | C_max_ (ng/mL) | AUC_0-24h_ (ng × hour/mL) |
| Midostaurin (n = 54) | 1.1 (1.1-3.1) | 2273.3 (710.3) | 25 657.5 (10,872.7) |
| CGP62221 (n = 51) | 3.1 (0.00-8.1) | 1882.0 (432.5) | 36 486.5 (9769.7) |
| CGP52421 (n = 45) | 3.1 (1.1-8.1) | 1248.6 (208.4) | 25 837.2 (4034.0) |

Values are mean (SD) except for T_max_, which are median (range).

C_max_, peak plasma concentration; T_max_, time to C_max_; AUC_0-12h_, area under the plasma concentration–time curve from time 0 to 12 hours; AUC_0-24h_, area under the plasma concentration–time curve from time 0 to 24 hours; SD, standard deviation.

###### Supplementary Table 4 Summary of Pharmacokinetic Parameters for Moxifloxacin (Day 3) (Pharmacokinetic Set)

| Statistics | T_max_ (hours) | C_max_ (ng/mL) | AUC_0-tlast_ (ng * hour/mL) |
| --- | --- | --- | --- |
| No. of participants | 43 | 43 | 43 |
| Mean | — | 2544.2 | 29 407.9 |
| SD | — | 495.0 | 5165.6 |
| CV% mean | — | 19.5 | 17.6 |
| Geometric mean | — | 2496.3 | 28 951.1 |
| CV% geometric mean |  | 20.1 | 18.3 |
| Median | 2.1 | 2460.0 | 29 802.4 |
| Range | 0.6-4.1 | 1650.0-3470.0 | 19 904.3–41 093.8 |

CV%, coefficient of variation (%) = Standard Deviation/Mean∙100.

CV% geo-mean = √ (exp (variance for log transformed data) -1)∙100.C_max_, peak plasma concentration; AUC_0-tlast_, area under the curve from time 0 to the last measurable concentration sampling time; T_max_, time to C_max_; SD, standard deviation; CV, coefficient of variation.

###### Supplementary Table 5 Adverse Events (All Grade 1/2) (Safety Set)

|  | Midostaurin (n = 79) | Moxifloxacin (n = 44) | Placebo (n = 68) | Total (N = 191) |
| --- | --- | --- | --- | --- |
| Participants with adverse events, no. (%) | 43 (54.4) | 9 (20.5) | 14 (20.6) | 66 (34.6) |
| Preferred term, no. (%) |  |  |  |  |
| Nausea | 30 (38.0) | 5 (11.4) | 1 (1.5) | 36 (18.8) |
| Diarrhea | 25 (31.6) | 1 (2.3) | 4 (5.9) | 30 (15.7) |
| Vomiting | 19 (24.1) | 1 (2.3) | 2 (2.9) | 22 (11.5) |
| Headache | 5 (6.3) | 4 (9.1) | 6 (8.8) | 15 (7.9) |
| Abdominal pain | 4 (5.1) | 0 (0.0) | 1 (1.5) | 5 (2.6) |
| Flatulence | 3 (3.8) | 1 (2.3) | 1 (1.5) | 5 (2.6) |
| Dizziness | 0 (0.0) | 1 (2.3) | 2 (2.9) | 3 (1.6) |
| Eructation | 3 (3.8) | 0 (0.0) | 0 (0.0) | 3 (1.6) |
| Dyspepsia | 1 (1.3) | 1 (2.3) | 0 (0.0) | 2 (1.0) |
| Flushing | 2 (2.5) | 0 (0.0) | 0 (0.0) | 2 (1.0) |
| Hiccups | 2 (2.5) | 0 (0.0) | 0 (0.0) | 2 (1.0) |
| Pollakiuria | 0 (0.0) | 1 (2.3) | 1 (1.5) | 2 (1.0) |
| Salivary hypersecretion | 2 (2.5) | 0 (0.0) | 0 (0.0) | 2 (1.0) |
| Tachycardia | 2 (2.5) | 0 (0.0) | 0 (0.0) | 2 (1.0) |
| Arthralgia | 0 (0.0) | 0 (0.0) | 1 (1.5) | 1 (0.5) |
| Dry mouth | 0 (0.0) | 0 (0.0) | 1 (1.5) | 1 (0.5) |
| Dysmenorrhea | 1 (1.3) | 0 (0.0) | 0 (0.0) | 1 (0.5) |
| Hot flushes | 1 (1.3) | 0 (0.0) | 0 (0.0) | 1 (0.5) |
| Rash | 0 (0.0) | 1 (2.3) | 0 (0.0) | 1 (0.5) |
| Sexually transmitted disease | 1 (1.3) | 0 (0.0) | 0 (0.0) | 1 (0.5) |
